# High prevalence of undiagnosed iron deficiency in endometriosis patients: A cross‐sectional study

**DOI:** 10.1002/ijgo.15994

**Published:** 2024-11-20

**Authors:** Hanna R. Goldberg, Carmen McCaffrey, Jonathon Solnik, Nucelio Lemos, Mara Sobel, Sari Kives, A. Kinga Malinowski, Nadine Shehata, John Matelski, Klaudia Szczech, Ally Murji

**Affiliations:** ^1^ Department of Obstetrics and Gynecology University of Toronto Toronto Ontario Canada; ^2^ Department of Obstetrics and Gynecology St. Michael's Hospital Toronto Ontario Canada; ^3^ Department of Obstetrics and Gynecology Mount Sinai Hospital Toronto Ontario Canada; ^4^ Department of Medicine Mount Sinai Hospital Toronto Ontario Canada; ^5^ Department of Medicine University of Toronto Toronto Ontario Canada; ^6^ Department of Obstetrics and Gynecology Institute for Better Health, Trillium Health Partners Mississauga Ontario Canada

**Keywords:** anemia, endometriosis, ferritin, iron deficiency, quality of life, transferrin saturation

## Abstract

**Objective:**

The primary objective was to evaluate the prevalence of undiagnosed iron deficiency in patients with endometriosis.

**Methods:**

We performed a multi‐center, cross‐sectional study at two tertiary care hospitals. We included 251 non‐pregnant women (18–50 years old) presenting with a clinical or surgical diagnosis of symptomatic endometriosis. Patients who consented to the study underwent screening bloodwork (including complete blood count, ferritin, and transferrin saturation) and completed the study survey assessing demographics, medical and surgical history, and validated questionnaires to assess iron deficiency and endometriosis symptoms.

**Results:**

The prevalence of iron deficiency in our endometriosis cohort was 53.4% (134/251), and the prevalence of iron deficiency anemia was 13.5% (34/251). Patients with iron deficiency were more likely to have heavy menstrual bleeding (HMB) compared with patients without iron deficiency (66/133, 49.6% vs. 40/115, 34.8%, *p* = 0.022). Nonetheless, 58% (142/251) of our study population did not endorse HMB. Despite absence of HMB, 47% (67/142) of these patients were iron‐deficient. Transferrin saturation was diagnostic for iron deficiency in 63 of 176 patients (35.7%) who had a normal ferritin (≥30 ng/mL). Patients with iron deficiency had a significantly lower adjusted median Functional Assessment of Chronic Illness Therapy Fatigue Subscale score compared with those without iron deficiency (26.3. vs. 29.8, *p* = 0.025).

**Conclusion:**

This study highlights the high prevalence of iron deficiency, which remains undiagnosed in over half of patients with endometriosis presenting to a gynecologist. Future research should focus on assessing the effectiveness of iron therapy in improving symptoms and overall well‐being in this population.

## Synopsis

This study highlights the high prevalence of iron deficiency, which remains undiagnosed in over half of patients with endometriosis presenting to a gynecologist.

## 1 INTRODUCTION

Iron deficiency (ID) and iron deficiency anemia (IDA) contribute a significant disease burden worldwide.^1^, ^2^, ^3^ Globally, there were over 1.2 billion cases of IDA in 2016.^2^ Symptoms of ID include fatigue, weakness, dizziness, irritability, decreased stamina, dyspnea, sleep disturbances, poor concentration, and depression.^4^, ^5^, ^6^, ^7^, ^8^ Interestingly, there is a striking similarity between these symptoms and those associated with endometriosis.

Endometriosis is a common condition characterized by pain and infertility.^9^, ^10^, ^11^ It is known to have a profound negative impact on quality of life.^9^, ^10^, ^11^, ^12^ Fatigue is a well characterized and common symptom of endometriosis.^13^, ^14^ The prevalence of fatigue in the endometriosis population is significantly higher compared to the general female population.^14^, ^15^ The similarity in symptoms reported by patients with endometriosis and ID raise the question of whether symptoms such as fatigue, depression, and sleep disturbance in patients with endometriosis are related to the disease itself, or whether there is a contribution from another underlying mechanism, such as ID/IDA.

Our primary objective was to evaluate the prevalence of undiagnosed ID in patients with endometriosis. Secondary objectives included evaluating the prevalence of IDA in patients with endometriosis and evaluating symptoms of ID and endometriosis in this population using validated patient‐reported outcome tools.

## 2 MATERIALS AND METHODS

### 2.1 Patient population

We performed a multi‐center, cross‐sectional study at two tertiary care hospitals. We included non‐pregnant women between the ages of 18 and 50 years presenting with a clinical or surgical diagnosis of symptomatic endometriosis. The diagnosis of endometriosis, whether clinical or surgical, was determined by the patient's gynecologist with a sub‐specialization in minimally invasive gynecology and was confirmed with medical chart review. Patients were excluded if they had a previous known diagnosis of ID/IDA over the past year (self‐reported or identified in medical chart), were treated with oral or intravenous iron within the past year, were less than 6 months postpartum or postoperative from any major abdominal or pelvic surgery, were postmenopausal, had symptoms attributed to uterine fibroids as determined by the gynecologist, had a history of medical treatment of uterine fibroids over the past 2 years, or were unable to read or understand English. Eligible patients presenting to gynecologic outpatient clinics were approached to participate in the study. This study received Research Ethics Board approval by Mount Sinai Hospital (21‐0149‐E) and St Michael's Hospital (21‐302‐C).

### 2.2 Intervention

Patients who provided informed consent to the research study underwent screening bloodwork (including complete blood count, ferritin, and transferrin saturation [TSAT]) and completed the study survey assessing demographics, and medical and surgical history. The presence of heavy menstrual bleeding (HMB) was ascertained based on four questions, including the need to double up on pads/tampons, changing pads/tampons every hour, waking up at night to change pads/tampons, and avoiding activities because of fear of heavy flow. Patients were classified as having HMB if they answered positively to one or more questions. Furthermore, the study survey included the following validated questionnaires:

- *Endometriosis Health Profile‐5* (*EHP*‐*5*)—A validated tool, scored on a scale of 0 to 100, that assesses the impact of endometriosis on health‐related quality of life (Data S1).^16^ A higher score indicates a lower quality of life.
- *Functional Assessment of Chronic Illness Therapy*—*Fatigue Subscale* (*FACIT‐FS*) (Data S1)—This tool is scored on a scale from 0 to 52 with higher scores indicating better quality of life. It has been validated in the HMB population.^17^
- *Functional Assessment of Cancer Therapy‐Anemia Subscale* (*FACT‐An Subscale*) (Data S1)—This 20‐item anemia subscale comprises 13 items that assess fatigue (FACIT‐FS) as well as seven additional symptoms associated with anemia (e.g., shortness of breath, headache, dizziness).^18^ It is scored on a scale from 0 to 80, with higher scores indicating better quality of life. This questionnaire has been validated in populations without cancer.
- *Short Form 36* (*SF‐36*) *Vitality Subscale* (VS) (Data S1)—This four‐item questionnaire that was designed to measure fatigue in healthy individuals in the general population has also been validated in individuals with chronic illness in the form of scleroderma.^19^ It is scored on a scale of 0–100, with a higher score indicating lower fatigue.

### 2.3 Outcome

The primary outcome was the prevalence of undiagnosed ID, defined as ferritin <30 ng/mL or TSAT <20%. Secondary outcomes included: (1) the prevalence of IDA in patients with endometriosis, defined as hemoglobin <12.0 g/dL and ferritin <30 ng/mL or TSAT <20%; (2) difference in ID symptoms between ID and non‐ID groups using the FACIT‐FS, FACT‐AN, and SF‐36 VS scales; and (3) difference in endometriosis symptoms between ID and non‐ID groups using the EHP‐5.

Ferritin is a highly sensitive measure of ID.^20^ However, given that it is an acute‐phase reactant, it may be elevated in certain inflammatory conditions.^20^, ^21^ There is significant variability in the literature regarding threshold values for ID. Mast et al. (1998) found that a threshold of ferritin <12 ng/mL was highly specific for ID (100%) but it was only 73% sensitive. In contrast, an upper diagnostic value of 30 ng/mL provides a sensitivity of 96% and still maintains a specificity of 100%.^21^ As such, the cut‐off value for ferritin used in the current study was set at 30 ng/mL. Given the potential insensitivity of ferritin to identify ID in inflammatory states, and given the inflammatory milieu underpinning endometriosis, an additional indicator of ID was added in the form of TSAT, a more specific measure of ID, where TSAT <20% is diagnostic of ID.^20^, ^22^

Other markers of ID, including serum iron and total iron‐binding capacity (TIBC), were not used in this study. TSAT is the ratio of serum iron to TIBC expressed as a percentage. An individual with ID will usually exhibit a low concentration of serum iron, and serum iron measurement is needed to allow calculation of the percentage of TSAT; however, according to the British Society of Hematology guidelines, measuring serum iron in isolation is not helpful, as it is a dynamic parameter with well‐established day‐to‐day variability.^23^

### 2.4 Statistical methods and sample size calculation

Continuous variables were summarized using median and interquartile range. Categorical variables were summarized using frequencies and percentages. Unadjusted comparisons of baseline characteristics were performed using either chi‐squared test, Wilcoxon rank sum test, or *t*‐test, as appropriate. Prevalence was calculated by reporting a proportion of patients meeting the diagnostic criteria in relation to the total number of patients screened. Assuming a population prevalence of 20%, a sample size of 246 is sufficient for ±5% precision in the estimated prevalence, with 95% confidence. For adjusted outcome analyses, we used linear regression to compare scores on the EHP‐5, FACIT‐FS, FACT‐An, and SF‐36 between ID and IDA groups. The following covariates were used for adjustment in the models: age, body mass index (BMI, calculated as weight in kilograms divided by the square of height in meters), ethnicity, smoking status, superficial versus deep endometriosis, presence of fibroids, and presence of endometrioma.

As HMB can be on the causal pathway between ID (exposure) and quality of life outcomes, we performed a secondary analysis where we explored HMB as a possible mechanism for the influence of ID on the quality‐of‐life outcomes, using R package mediation.^24^ Both the mediator and outcome models were adjusted for age, BMI, smoking status, race, clinical/surgical diagnosis, and endometriosis medication use. We estimated the average causal mediation effect (ACME) of ID via HMB, the average direct effect (ADE) of ID, and the proportion of the total effect of ID mediated by HMB, along with quasi‐Bayesian 95% confidence intervals and *p*‐values.

We adopted alpha = 0.05 as the threshold for statistical significance. Statistical analyses were performed using R version 4.2.1.^25^

## 3 RESULTS

We approached 265 consecutive patients to participate, of whom 251 consented and completed the study (94.7%). The prevalence of ID in our endometriosis cohort was 53.4% (134/251), and the prevalence of IDA was 13.5% (34/251). Baseline patient characteristics according to ID status are presented in Table 1. We found differences in smoking status and ethnicity between ID and non‐ID groups at baseline. Patients with ID were more likely to have HMB compared with patients without ID deficiency (66/133 [49.6%] vs. 40/115 [34.8%], *p* = 0.022).

**TABLE 1:** Baseline characteristics of study population.

	All patients (*n* = 251)	With iron deficiency (*n* = 134)	Without iron deficiency (*n* = 117)	*p*‐value
Age (y)	36 (31–41)	35 (31–42)	36 (31–40)	0.835
Gravidity	0 (0, 1)	0 (0, 1)	0 (0, 1)	0.880
Parity	0 (0, 1)	0 (0, 0)	0 (0, 0)	0.408
BMI	25.1 (21.5–30.0)	25.5 (21.9–30.7)	24.9 (21.4–28.3)	0.264
Smoking status				**0.025**
Never smoked	169 (68.1)	98 (74.2)	71 (61.2)	
Previously smoked	52 (21.0)	19 (14.4)	33 (28.4)	
Currently smoking	27 (10.9)	15 (11.4)	12 (10.3)	
Ethnicity				**0.026**
Black	18 (7.2)	11 (8.2)	7 (6.0)	
South Asian	35 (13.9)	25 (18.7)	10 (8.5)	
Caucasian	129 (51.4)	58 (43.3)	71 (60.7)	
Other	69 (27.5)	40 (29.9)	29 (24.8)	
Diagnosis of endometriosis				0.120
Surgical	95 (38.0)	57 (42.9)	38 (32.5)	
Clinical	156 (62.3)	77 (57.5)	79 (67.5)	
Endometriosis severity				0.510
Superficial	159 (63.3)	88 (65.7)	71 (60.7)	
Deep	92 (37.4)	46 (35.1)	46 (40.0)	
Endometrioma on imaging	153 (64.0)	84 (67.2)	69 (60.5)	0.348
Adenomyosis on imaging	54 (23.4)	32 (25.8)	22 (20.6)	0.433
Concomitant fibroids on imaging	47 (20.3)	25 (20.2)	22 (20.6)	1.000
Previous endometriosis surgery	89 (35.6)	54 (40.6)	35 (29.9)	0.103
Menses in the last 3 months				0.075
Yes	173 (70.3)	99 (75.6)	74 (64.3)	
Heavy menstrual bleeding	106 (42.2)	66 (49.6)	40 (34.8)	**0.022**
Endometriosis‐specific medications in last 3 months				0.561
0	125 (50.6)	67 (50.0)	60 (51.3)	
1	114 (45.4)	60 (44.8)	54 (46.2)	
2+	10 (4.0)	7 (5.2)	3 (2.6)	

*Note*: All values are represented as median (interquartile range) or frequency (%).

Abbreviation: BMI, body mass index (calculated as weight in kilograms divided by the square of height in meters).

Table 2 shows differences in hematologic parameters between groups. ID patients had significantly lower hemoglobin, ferritin, and TSAT levels. We also evaluated hematologic parameters in the 176 patients who had ferritin ≥30 ng/mL. In this group of patients, TSAT was diagnostic for ID in 63 of 176 patients (35.8%). This indicates that these individuals would not have been diagnosed with ID based on ferritin alone.

**TABLE 2:** Hematologic parameters between iron‐deficient and non‐iron‐deficient groups.

	All patients (*n* = 251)	With iron deficiency (*n* = 134)	Without iron deficiency (*n* = 117)	*p‐*value
Hemoglobin (g/dL)	13.2 (12.5–13.8)	12.9 (12.1–13.6)	13.4 (12.9–13.9)	**<0.001**
Ferritin (ng/mL)	51 (29–72)	29 (21–55.25)	65 (51–93)	**<0.001**
Transferrin saturation (%)	21 (14–29)	14 (11–18)	29 (24–33)	**<0.001**

*Note*: All values are represented as median (interquartile range).

Forty‐two percent of our population endorsed HMB (106/251). In the subgroup of patients with HMB, 62% (66/106) had ID. Conversely, 58% (142/251) of our population did not endorse HMB. Of the patients in this subgroup, 47% (67/142) were iron‐deficient.

Table 3 shows adjusted scores for symptom severity and quality of life indicators in patients with and without ID. Patients with ID had a significantly lower median FACIT‐FS score than those without ID (26.3. vs. 29.8, *p* = 0.025). There was no difference in the adjusted EHP‐5, FACT‐An scores, and SF‐36 VS scores between ID and non‐ID groups. Based on the mediation analysis, 13% of the effect of ID on FACIT was attributable to HMB; however, this effect was not statistically significant (0.100).

**TABLE 3:** Adjusted comparisons of symptoms and quality of life scores.

	With iron deficiency	Without iron deficiency	*p‐*value
Dysmenorrhea	7.48 (5.5–9.5)	7.2 (5.1–9.2)	0.486
Non‐menstrual pelvic pain (VAS)	5.6 (3.7–7.4)	6.2 (4.2–8.1)	0.176
Dyspareunia	5.4 (3.3–7.6)	4.6 (2.4–6.9)	0.104
EHP‐5 score	49.1 (33.6–64.5)	47.3 (31.3–63.2)	0.619
FACT‐An score	44.9 (36.0–53.9)	48.9 (39.6–58.2)	0.062
FACIT‐Fatigue score	26.3 (19.6–32.9)	29.8 (22.9–36.7)	**0.025**
SF‐36 VS score	41.5 (27.6–55.4)	39.3 (25.0–53.5)	0.471

*Note*: All values are represented as median (interquartile range).

Those values were bolded to demonstrate that they are statistically significant (*p*<0.05).

Abbreviations: EHP, endometriosis health profile; FACIT‐FS, functional assessment of chronic illness therapy—fatigue subscale; FACT‐an subscale, functional assessment of cancer therapy‐anemia subscale; SF‐36, Short Form 36; VAS, visual analog scale; VS, vitality subscale.

## 4 DISCUSSION

### 4.1 Principal findings

This cross‐sectional study conducted at two tertiary care centers in Canada showed a high prevalence of ID in patients presenting with endometriosis, with undiagnosed ID noted in more than half (53.4%) of the participants. A significant proportion (13.5%) of these patients had IDA. These results suggest that ID is a common comorbidity in patients with endometriosis and that these patients are more likely to experience decreased quality of life and fatigue. HMB did not significantly mediate/impact the effect of ID on quality of life. Over half of our population did not suffer from HMB, yet the prevalence of undiagnosed ID in this subgroup remained close to half (47%). The study also highlighted the challenges associated with diagnosing ID in the context of endometriosis. Using ferritin as the sole diagnostic marker of ID may be problematic, as it is an acute‐phase reactant and can be influenced by inflammatory conditions, potentially leading to false‐negative results. In our study, the median ferritin level in the ID group was just below the ID threshold of 30 ng/mL, indicating the potential limitations of relying solely on ferritin levels for diagnosing ID in the context of endometriosis. In fact, 35.8% of ID would have been missed if ferritin was the sole test used. Our findings support the inclusion of additional markers, such as TSAT, for accurate evaluation of ID in patients with endometriosis.

### 4.2 Relation to other studies

The prevalence of ID in our study population of patients with endometriosis is considerably higher than what has been previously reported in the general population. In the *Iron Sufficiency of Canadians* report, Statistics Canada observed that in 2012, the prevalence rates of ID and IDA in women aged 20–49 were 9% and 3.7%, respectively.^26^ It is important to note that the ferritin reference value used in their study was ≤15 ng/mL, whereas the threshold used in our study was <30 ng/mL.

In our study population, the median ferritin level in the ID group was 29 ng/mL, which is just below the ID threshold (30 ng/mL). A low TSAT level (<20%) was more specific and diagnostic of ID. In early stages of ID, the TSAT level may actually still be normal.^20^ TSAT is a marker of iron stores that decreases after ferritin decreases.^20^ When both TSAT and ferritin are below the normal reference ranges, this indicates a state of absolute ID.^20^ In patients with inflammatory conditions, ferritin may be normal or elevated as it is an acute‐phase reactant.^20^ This is consistent with the findings of our study and highlights the need for the use of TSAT in combination with ferritin for evaluating ID in patients with endometriosis.^20^

There are three pathophysiologic mechanisms that can explain the high prevalence of ID that was found in our study population. First, up to 68% of endometriosis patients suffer from HMB, likely due to an overlap with adenomyosis.^14^ In our study population, 23.4% had concomitant adenomyosis demonstrated on ultrasound and 42.2% reported HMB. As expected, there was a higher prevalence of ID in those with HMB (62.3%) than in those without HMB (47.2%). However, this does not explain the high prevalence of ID in those without HMB. Second, proinflammatory cytokines and inflammatory cells present in endometriosis which can lead to dysregulation of the hepcidin and ferroportin 1 pathway, which are involved in intestinal iron absorption.^27^, ^28^ Lastly, there is a high likelihood of undiagnosed comorbidity between endometriosis and several autoimmune diseases, including inflammatory bowel disease, which may impair iron absorption.^29^

The present study provides an insight into the high prevalence of ID, which remains undiagnosed in over half of patients with endometriosis presenting to a gynecologist. The impact of ID on symptoms and quality of life in endometriosis patients warrants further investigation, though we found that ID is associated with fatigue, a common symptom experienced by endometriosis patients. Similarly, Ramin‐Wright et al. showed that fatigue was experienced by over half of patients diagnosed with endometriosis (50.7% vs. 22.4%, *p* < 0.001).^13^ In a survey of over 26 000 women with a self‐reported diagnosis of endometriosis utilizing validated questionnaires, over 53% reported fatigue, which resulted in significant productivity impairments.^14^ Future research should focus on assessing the effectiveness of iron therapy (oral and/or intravenous) in improving symptoms, specifically fatigue, and overall well‐being in this population.

### 4.3 Strengths and weaknesses of the study

The strengths of this study include its multi‐center study design and relatively large sample size that was consecutively recruited. By excluding patients with any suggestion of a known diagnosis of ID/IDA, we were able to ascertain a realistic prevalence of undiagnosed ID in our population. However, our findings must be interpreted in the context of the cross‐sectional study design, which precludes the use of matched controls, the assessment of causal effects, and potentially limits the generalizability to all populations. Nonetheless, the cosmopolitan make‐up of our city is reflected in the diversity of ethnicities seen in our patient population, which is reassuring with respect to the latter. Furthermore, the use of self‐administered questionnaires, especially those related to menses and bleeding patterns, is subject to recall bias. To decrease the contribution of bias, we used validated questionnaires whenever possible. Finally, with the exception of quality of life measured on FACIT‐FS, which evaluates self‐reported fatigue and its impact upon daily activities and function, we did not find any differences in other quality‐of‐life measures. This may be because ID does not affect the of quality‐of‐life domains evaluated by the other scales, or because the study was simply not powered to detect these changes. Although ID did not affect other quality‐of‐life measures, the impact of anemia on these outcomes was not explored.

## 5 CONCLUSION

Our study highlights the high prevalence of ID in the endometriosis population. Importantly, ID remains undiagnosed in over half of patients with endometriosis presenting to a gynecologist. Given that endometriosis is an inflammatory condition, ferritin alone may be less reliable in diagnosing ID. The use of TSAT to accurately diagnose ID in this population is important. Further investigation into whether treatment of ID improves quality of life in endometriosis patients should be a priority.

## AUTHOR CONTRIBUTIONS

All authors contributed significantly to conception and design of the study. AM, HG, CM, JS, MS, NL, and SK contributed to patient recruitment. AM, HG, CM, and KS contributed significantly to data collection. AM, HG, and JM significantly contributed to data analysis, statistical analysis, and data interpretation. AM and HG prepared the initial draft of the manuscript and all authors significantly contributed to critically revising the manuscript.

## FUNDING INFORMATION

This research was funded by Mount Sinai Department of Obstetrics and Gynecology and University of Toronto Department of Obstetrics and Gynecology. This study was also funded by the CanSAGE Research Grant. The funders had no role in the design of the study or collection, analysis and interpretation of the data, writing of the report or the decision to submit the article for publication.

## CONFLICT OF INTEREST STATEMENT

AKM: Pfizer, Leo Pharma, Alexion (honoraria), Pfizer (consultancy); AM: advisory board and speaker bureau (Abbvie, Bayer, Pfizer).

## Supporting information


Data S1.

## Data Availability

The data that support the findings of this study are available on request from the corresponding author. The data are not publicly available due to privacy or ethical restrictions.
