# Supplementary material for: High prevalence of undiagnosed iron deficiency in endometriosis patients: A cross‐sectional study
Source: Int J Gynaecol Obstet. 2024 Nov 20;168(3):1321–7. doi: 10.1002/ijgo.15994 (PMC11823345; doi:10.1002/ijgo.15994)
Supplement: Supplementary file 1 — Data S1. [file IJGO-168-1321-s001.docx]

**Supplemental Material**

Table 1. Endometriosis Health Profile-5 (EHP-5)

During the last 4 weeks, how often, because of your endometriosis, have you… (check the appropriate box)

|  | **Never** | **Rarely** | **Sometimes** | **Often** | **Always** |
| --- | --- | --- | --- | --- | --- |
| **Found it difficult to walk because of pain?** |  |  |  |  |  |
| **Felt as though your symptoms are ruling your life?** |  |  |  |  |  |
| **Had mood swings?** |  |  |  |  |  |
| **Felt others do not understand what you are going through?** |  |  |  |  |  |
| **Felt your appearance has been affected?** |  |  |  |  |  |

Table 2. Functional Assessment of Chronic Illness Therapy – Fatigue Subscale (FACIT-FS)

Please answer these questions as it applies to the **past 7 days**

|  | **Not at all** | **A little bit** | **Somewhat** | **Quite a bit** | **Very much** |
| --- | --- | --- | --- | --- | --- |
| **I feel fatigued** |  |  |  |  |  |
| **I feel weak all over** |  |  |  |  |  |
| **I feel listless (“washed out”)** |  |  |  |  |  |
| **I feel tired** |  |  |  |  |  |
| **I have trouble starting things because I am tired** |  |  |  |  |  |
| **I have trouble finishing things because I am tired** |  |  |  |  |  |
| **I have energy** |  |  |  |  |  |
| **I am able to do my usual activities** |  |  |  |  |  |
| **I need to sleep during the day** |  |  |  |  |  |
| **I am too tired to eat** |  |  |  |  |  |
| **I need help doing my usual activities** |  |  |  |  |  |
| **I am frustrated by being too tired to do the things I want to do** |  |  |  |  |  |
| **I have to limit my social activity because I am tired** |  |  |  |  |  |

Table 3. Functional Assessment of Cancer Therapy-Anemia Subscale (FACT-An Subscale)

Please answer these questions as it applies to the **past 7 days**

|  | **Not at all** | **A little bit** | **Somewhat** | **Quite a bit** | **Very much** |
| --- | --- | --- | --- | --- | --- |
| **I feel fatigued** |  |  |  |  |  |
| **I feel weak all over** |  |  |  |  |  |
| **I feel listless (“washed out”)** |  |  |  |  |  |
| **I feel tired** |  |  |  |  |  |
| **I have trouble starting things because I am tired** |  |  |  |  |  |
| **I have trouble finishing things because I am tired** |  |  |  |  |  |
| **I have energy** |  |  |  |  |  |
| **I have trouble walking** |  |  |  |  |  |
| **I am able to do my usual activities** |  |  |  |  |  |
| **I need to sleep during the day** |  |  |  |  |  |
| **I feel lightheaded (dizzy)** |  |  |  |  |  |
| **I get headaches** |  |  |  |  |  |
| **I have been short of breath** |  |  |  |  |  |
| **I have pain in my chest** |  |  |  |  |  |
| **I am too tired to eat** |  |  |  |  |  |
| **I am interested in sex** |  |  |  |  |  |
| **I am motivated to do my usual activities** |  |  |  |  |  |
| **I need help doing my usual activities** |  |  |  |  |  |
| **I am frustrated by being too tired to do the things I want to do** |  |  |  |  |  |
| **I have to limit my social activity because I am tired** |  |  |  |  |  |

Table 4. Short Form 36 (SF-36) Vitality Subscale (VS)

For each question, please give the answer that comes closest to how you have been feeling in the **last 4 weeks**

|  | **All of the time** | **Most of the time** | **A good bit of the time** | **Some of the time** | **A little of the time** | **None of the time** |
| --- | --- | --- | --- | --- | --- | --- |
| **Did you feel full of pep?** |  |  |  |  |  |  |
| **Did you have a lot of energy?** |  |  |  |  |  |  |
| **Did you feel worn out?** |  |  |  |  |  |  |
| **Did you feel tired?** |  |  |  |  |  |  |
